# Neurofibromatosis Type 2 Tumor Suppressor Protein, NF2, Induces Proteasome-Mediated Degradation of JC Virus T-Antigen in Human Glioblastoma

**DOI:** 10.1371/journal.pone.0053447

**Published:** 2013-01-07

**Authors:** Sarah Beltrami, Emanuela Branchetti, Ilker K. Sariyer, Jessica Otte, Michael Weaver, Jennifer Gordon

**Affiliations:** 1 Department of Neuroscience and Center for Neurovirology, Temple University School of Medicine, Philadelphia, Pennsylvania, United States of America; 2 Biomedical Neuroscience Graduate Program, Temple University School of Medicine, Philadelphia, Pennsylvania, United States of America; 3 Department of Neurosurgery, Temple University School of Medicine, Philadelphia, Pennsylvania, United States of America; The University of North Carolina at Chapel Hill, United States of America

## Abstract

Neurofibromatosis type 2 protein (NF2) has been shown to act as tumor suppressor primarily through its functions as a cytoskeletal scaffold. However, NF2 can also be found in the nucleus, where its role is less clear. Previously, our group has identified JC virus (JCV) tumor antigen (T-antigen) as a nuclear binding partner for NF2 in tumors derived from JCV T-antigen transgenic mice. The association of NF2 with T-antigen in neuronal origin tumors suggests a potential role for NF2 in regulating the expression of the JCV T-antigen. Here, we report that NF2 suppresses T-antigen protein expression in U-87 MG human glioblastoma cells, which subsequently reduces T-antigen-mediated regulation of the JCV promoter. When T-antigen mRNA was quantified, it was determined that increasing expression of NF2 correlated with an accumulation of T-antigen mRNA; however, a decrease in T-antigen at the protein level was observed. NF2 was found to promote degradation of ubiquitin bound T-antigen protein via a proteasome dependent pathway concomitant with the accumulation of the JCV early mRNA encoding T-antigen. The interaction between T-antigen and NF2 maps to the FERM domain of NF2, which has been shown previously to be responsible for its tumor suppressor activity. Co-immunoprecipitation assays revealed a ternary complex among NF2, T-antigen, and the tumor suppressor protein, p53 within a glioblastoma cell line. Further, these proteins were detected in various degrees in patient tumor tissue, suggesting that these associations may occur *in vivo*. Collectively, these results demonstrate that NF2 negatively regulates JCV T-antigen expression by proteasome-mediated degradation, and suggest a novel role for NF2 as a suppressor of JCV T-antigen-induced cell cycle regulation.

## Introduction

Disruption of the Neurofibromatosis type 2 (*NF2)* gene, on human chromosome 22, leads to the development of the autosomal dominant disorder Neurofibromatosis type 2, characterized by the development of schwannomas, meningiomas, and ependymomas [1]. Though these tumors are typically benign, NF2 loss has been implicated in the progression of many malignant tumors including highly aggressive mesothelioma tumors in humans [2], [3] and mice with heterozygous loss of NF2 are prone to the formation of multiple highly metastatic tumors, predominantly osteosarcomas and fibrosarcomas [4]. Loss of NF2 in glial cultures can lead to hyperproliferation, and formation of tumors in animal models [5]–[7]. Expression of NF2 has been shown to be absent in certain human glioblastomas and reintroduction can greatly suppress their growth [8]–[10].

The NF2 tumor suppressor protein is a member of the ERM family of proteins, which primarily facilitate cell to cell adhesion [11]–[13]. NF2 is responsive to cell confluence and growth factor availability [14]–[16]. Consequently, NF2 aides in the maintenance of contact inhibition of cell growth and anchorage dependence, whereby loss of NF2 facilitates invasion and mobility of transformed cells [6], [17], [18], [28]. NF2 is different from prototypical tumor suppressor proteins, such as p53, in that it is not known to directly affect the cell cycle, but rather acts as a crucial link between the extracellular environment and cell signaling pathways [14], [19]. Theories on the tumor suppressive roles of NF2 have largely been restricted to its actions as a scaffolding protein, however NF2 can be found in the nucleus, where its nuclear localization appears to be mediated by the cell cycle [20]–[22].

Previously, we have discovered the interaction of NF2 with the major regulatory protein of the human polyomavirus, JC virus (JCV), large tumor antigen (T-antigen), in malignant peripheral nerve sheath tumors (mpnsts) derived from JCV T-antigen transgenic mice [23]. JCV persistently infects the majority of individuals worldwide and is the causative agent of the rare but fatal demyelinating disease, progressive multifocal leukoencephalopathy, PML [24]. In addition to its role in PML pathogenesis, JCV has exhibited oncogenic potential in cell culture and experimental animal models where T-antigen expression leads to a broad range of CNS malignancies, most notably the formation of neuronal and glial origin tumors including medulloblastomas, astrocytomas, and primitive neuroectodermal tumors. JCV DNA or T-antigen protein expression has been detected in a similarly broad range of human tumors including medulloblastomas, astrocytomas, ependymomas, as well as CNS lymphomas and tumors of the gastrointestinal tract [25].

Potential mechanisms of JCV T-antigen induced oncogenesis have focused on the ability of T-antigen to disrupt the activity of cell cycle regulatory proteins including p53 and Rb [26], T-antigen's enhancement of oncogenic signaling through the Wnt pathway by stabilizing key members, such as β-catenin, LEF-1, and *c-myc* [27], and its interaction with DNA repair proteins [28], [29], to name a few. Surprisingly, few mechanisms have been identified that can repress T-antigen-mediated oncogenesis and attention has mainly focused on transcriptional regulation of the JCV promoter (for review see [25]). In addition, many polyomaviruses, including JCV, encode a conserved miRNA targeting the viral early mRNA as an autoregulatory mechanism [30]. Other potential negative regulators of T-antigen expression include SF2/ASF, which inhibits alternative splicing of the JCV early transcript [31], and CBP which partners with p53 to enhance acetylation of T-antigen, affecting its stability [32], [33].

In this study, we provide a new mechanism of tumor suppression by NF2 and demonstrate inhibition of JCV T-antigen expression. Here, we show that NF2, specifically its FERM domain, negatively regulates JCV T-antigen protein levels via a proteasome-mediated pathway in U-87 MG human glioblastoma cells. Our findings also extend to clinically obtained human glioblastomas, where T-antigen, NF2, and p53, can be detected. Taken together, these results provide new insights into the function of NF2, and provide a potential pathway for inhibiting T-antigen function in glial cells.

## Results

### NF2 Suppresses T-antigen Protein Expression and JCV Promoter Activity

**Figure 1:**
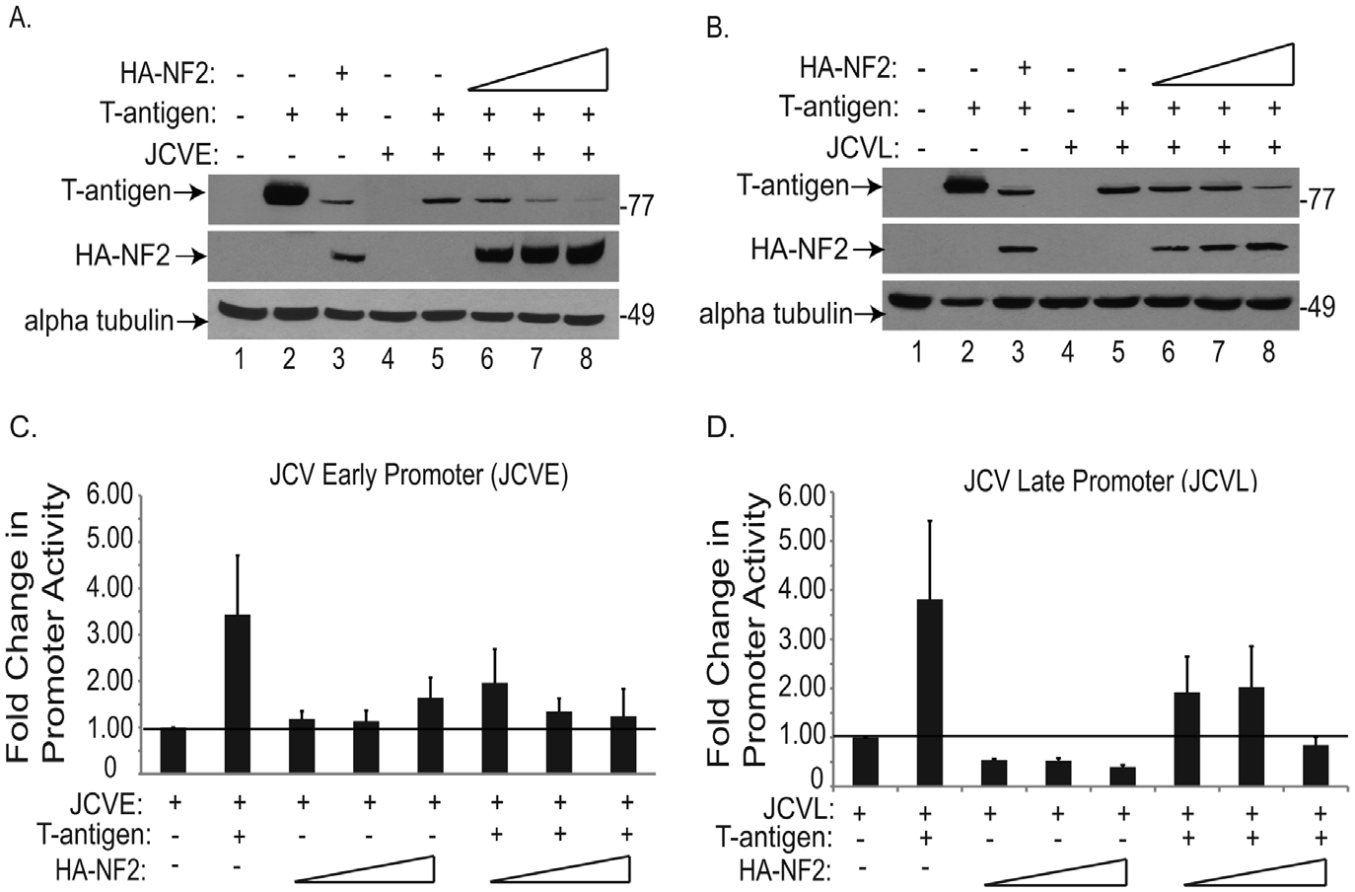
NF2 suppresses T-antigen protein expression and T-antigen mediated activation of JCV_E_ and JCV_L_ promoters. U-87 MG glioblastoma cells were transiently transfected with luciferase reporter constructs encoding either the JCV**_E_** (a, c) or JCV**_L_** (b, d) promoters, and plasmids encoding JCV T-antigen or HA tagged NF2 (HA-NF2). Immunoblotting of protein lysates revealed a dose-dependent decline in the expression of T-antigen, in the presence of increasing amounts of HA-NF2 (a, b lanes 5–8). Extracts were also used to conduct luciferase assays to determine JCV**_E_** (c) or JCV**_L_** (d) activation in response to these various transfection conditions. Results are shown here as fold change in luciferase activity, relative to the activity of the promoter alone. NF2 does not appreciably affect the activities of either promoter, while T-antigen greatly enhances the activity of both promoters. In combination, NF2 does prevent T-antigen from enhancing the activities of JCV**_E_** and JCV**_L_**.

We previously reported that NF2 colocalizes with the JCV early regulatory protein, T-antigen, in the nucleus of malignant peripheral nerve sheath tumors found in JCV T-antigen transgenic mice [23]. Other tumor suppressor proteins, such as p53 and Rb, also share T-antigen as a nuclear binding partner. Typically these interactions inhibit p53 and Rb from acting as negative regulators of the cell cycle. However, p53 can also act as a negative regulator of JCV replication, via its interaction with the JCV promoter [34]. Here, we set out to examine if NF2 may have an effect on the expression of JCV T-antigen protein. To this end, human U-87 MG glioblastoma cells were transiently transfected with plasmids encoding JCV T-antigen and increasing amounts of HA tagged NF2 (HA-NF2). Immunoblotting of these cellular extracts revealed a dose-dependent decline of T-antigen protein expression occurring with increasing expression of NF2 (Figures 1A and 1B). However, when the reciprocal experiment was conducted in this cell culture system, NF2 expression was unaffected in the presence of increasing amounts of T-antigen (data not shown).

In parallel, we investigated the functional effect this decline of T-antigen protein would have on the activity of the JCV promoter. The JCV promoter is bidirectional, with the early promoter (JCV_E_) encoding the regulatory genes for large and small T-antigens, and the late promoter (JCV_L_) encoding capsid and accessory genes. Luciferase constructs containing the JCV promoter in either the early or late orientation were co-transfected into U-87 MG cells, along with expression plasmids for T-antigen and HA-NF2. The presence of increasing amounts of NF2 resulted in retention of basal promoter activity of the JCV_E_ promoter (Figure 1C) and minor suppression of JCV_L_ activity (Figure 1D). Since T-antigen activates its own promoter, supplementation with T-antigen enhanced the activity of both early and late promoters; however this effect was ameliorated in the presence of NF2.

To confirm whether the effect of NF2 on JCV promoter activity is due to the decrease in T-antigen levels rather than NF2 acting directly upon the viral promoter, we conducted a chromatin immunoprecipitation (ChIP) assay. For this assay, BSB8 cells, derived from a JCV T-antigen induced primitive neuroectodermal tumor in transgenic mice were utilized [35]. Immunoprecipitation of DNA:protein complexes with antibodies to HA-NF2 and T-antigen, followed by PCR amplification using primers specific for the JCV promoter, detected T-antigen in complex with the JCV promoter. However, HA-NF2 failed to precipitate this DNA region, indicating that NF2 does not directly bind the JCV promoter (data not shown). This result is consistent with a previous study whereby NF2 could not be detected in the chromatin containing fraction of the nucleus [22]. Cumulatively, this data suggests that NF2 downregulates JCV transcription via an indirect mechanism, by reducing T-antigen protein expression, so that T-antigen cannot subsequently enhance viral promoter activity.

### NF2 Facilitates the Accumulation of JCV T-antigen mRNA

**Figure 2:**
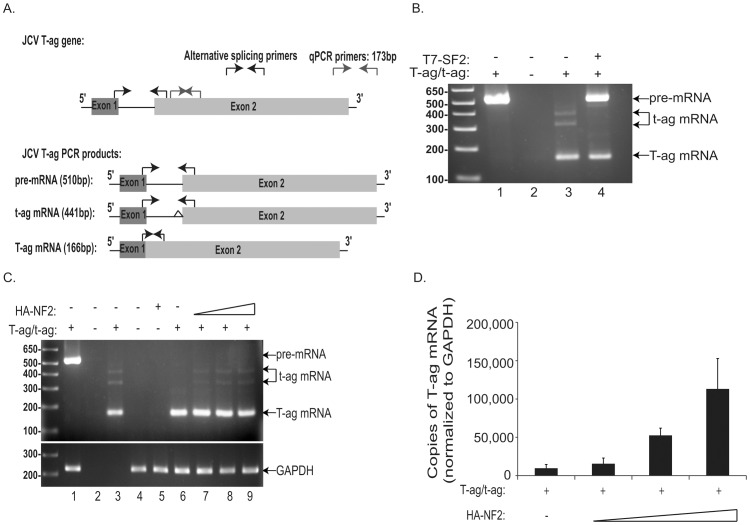
NF2 leads to accumulation of T-antigen mRNA, but does not affect its alternative splicing. Schematic showing all alternatively spliced forms of JCV T-antigen, including the pre-mRNA and both large and small T-antigen mRNAs, and location of primers used for PCR analysis are shown (A, black primers). PCR amplification of cDNA derived from T-antigen expressing BSB8 cells (B, lane 3), detected both large and small T-antigen mRNAs, but not pre-mRNA. Co-transfection of U-87 MG cells with expression plasmids for T-antigen and T7-SF2, an inhibitor of splicing, resulted in an accumulation of pre-mRNA and a reduction in large and small T-antigen mRNAs (B, lane 4). Co-transfection with T-antigen and increasing amounts of HA-NF2 plasmids did not alter the pattern of mRNAs detected (C, compare lanes 6 to 7–9). Q-PCR analysis was also conducted for T-antigen copy number (A, grey primers), expressed as copies of T-antigen mRNA per copy number of the control mRNA GAPDH*, with standard deviations of the mean shown here (D). This assay revealed a dose dependent accumulation of T-antigen mRNA in the presence of NF2. *Note that GAPDH primers used in Panel C only detect human and not mouse GAPDH, therefore a band representing GAPDH from the BSB8 mouse cell line was not detected (C, lane 3).

To date, only a few proteins are capable of suppressing the typically robust expression of JCV T-antigen in tumor cells. One of these proteins, p53, can suppress T-antigen expression through suppression of the viral promoter [34]. However, based on our findings above we ruled out the possibility of NF2 acting as a negative viral transcriptional regulator. As mentioned earlier, the alternative splicing factor, SF2, has been shown to inhibit the splicing of JCV T-antigen pre-mRNA thus reducing expression of both large and small T-antigens [36]. In order to determine if NF2 functions as an inhibitor of splicing, we utilized primers that can discriminate among JCV T-antigen pre-mRNA, and large and small T-antigen mRNAs, as previously described (Figure 2A, and [36]). We detected large and small T-antigen mRNAs, but not their shared pre-mRNA, in cDNA derived from JCV T-antigen expressing BSB8 cells (Figure 2B). As shown previously, T-antigen pre-mRNA accumulated with co-transfection of T7 tagged SF2 (T7-SF2) (Figure 2B, lane 4), but not with HA-NF2 in U-87 MG cells (Figure 2C, compare lanes 6–9), suggesting that the mechanism by which NF2 suppresses T-antigen protein occurs downstream of alternative splicing events. In addition, the relative amount of mature T-antigen mRNA in U-87 MG cells co-transfected with T-antigen and HA-NF2 was determined using large T-antigen specific primers (Figure 2A) by quantitative real time PCR (qRT-PCR) analysis. We observed a positive correlation between NF2 overexpression and T-antigen mature mRNA levels (Figure 2B). This accumulation of untranslated T-antigen mRNA combined with a reduction in T-antigen at the protein level as seen above (Figure 1) suggests a blockage of T-antigen expression by NF2 through post transcriptional intervention.

### The FERM Domain of NF2 is Responsible for its Binding and Degradation of T-antigen

**Figure 3:**
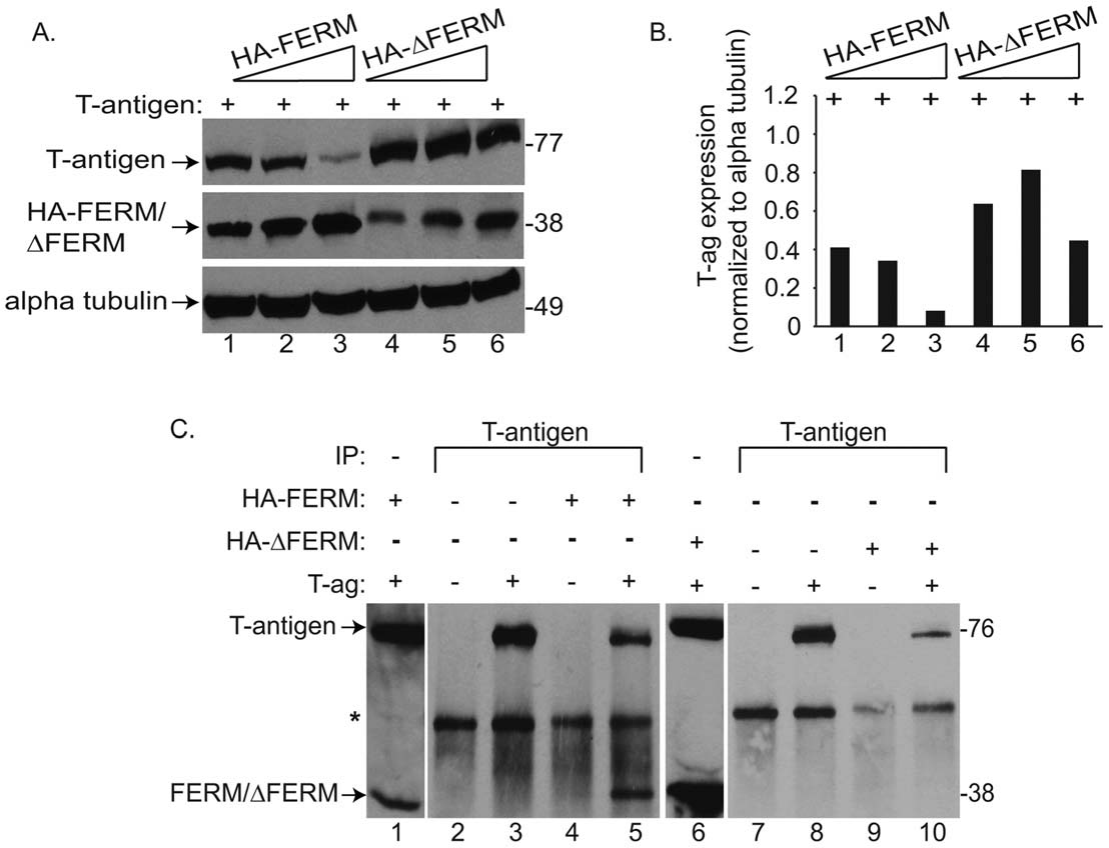
NF2 utilizes a proteasomal-dependent mechanism to promote the degradation of ubiquitin-bound T-antigen. To determine if NF2 utilizes the proteasome to enhance T-antigen protein turnover, U-87 MG cells, transfected with HA-NF2 and T-antigen, were treated with the general proteasome inhibitor, MG132 (A, right), or vehicle (A, left). As shown earlier (Figures 1A and 1B), enhanced expression of NF2, suppresses that of T-antigen (A, left top and bottom). However, this affect was abolished with the addition of MG132 (A, right top and bottom). Band intensity representing T-antigen expression was quantified, normalized to its loading control, and shown as fold change relative to lane 1 (A, bottom left and right). p21, a protein known to be subjected to proteasomal degradation, was immunoblotted for in extracts of untransfected U-87 MG cells or those transfected with HA-NF2 or T-antigen alone, exposed to increasing concentrations of MG132 (B). Expression of this protein increased with drug exposure (B, compare lanes 1, 4, and 7 to subsequent lanes), indicating the effectiveness of MG132 to inhibit degradation of proteins subjected to proteasomal degradation. The ability of T-antigen to bind to ubiquitin, a key post-translational protein attached to proteasome targeted proteins, was assessed by Co-IP. U-87 MG cells were transfected with combinations of Myc tagged p53 (Myc-p53), T-antigen, and HA tagged ubiquitin (HA-Ub), protein complexes were immunoprecipitated with an anti HA antibody or normal mouse serum. Subsequent immunoblotting demonstrated that p53 and T-antigen were not precipitated by the HA antibody alone (C, lanes 2 and 6), but did when co-transfected with HA-Ub (C, lanes 3–4 and 7). Collectively, these results provide the first evidence that T-antigen can bind ubiquitin and suggest that T-antigen is subjected to proteasome-mediated degradation.

To establish the region of NF2 responsible for its interactions with T-antigen, we utilized two truncations mutants of full length NF2, one containing the amino terminal FERM (Band 4.1, ezrin, radixin, moesin) domain (HA-FERM) and the other representing the C terminal half of the NF2 protein (HA-ΔFERM). The FERM domain of NF2 is responsible for its anchorage to the plasma membrane and actin binding [37]–[39]. Further, this domain is suggested to be the tumor suppressive region of NF2 in that it is necessary to prevent cellular proliferation in response to mitogenic signaling, as demonstrated by reductions in growth factor receptor signaling, MTT conversion, and colony formation in soft agar [22], [38], [40], [41]. Co-transfection of these NF2 mutants into U-87 MG cells along with T-antigen, revealed a dose-dependent decline in T-antigen levels with the HA-FERM mutant, similar to full length NF2 (Figure 3A, lanes 1–3), while T-antigen expression remained unaltered in the presence of the HA-ΔFERM mutant (Figure 3A, lanes 4–6). Further, co-immunopreciptation (Co-IP) assays in lysates from these cells revealed that T-antigen binds to the FERM, but not ΔFERM region of NF2 (Figure 3C, compare lanes 5 and 10). These studies further suggest that the FERM domain of NF2 is required for its tumor suppressive capabilities, specifically in T-antigen mediated malignancies.

### NF2 Promotes Proteasomal-mediated Degradation of T-antigen

**Figure 4:**
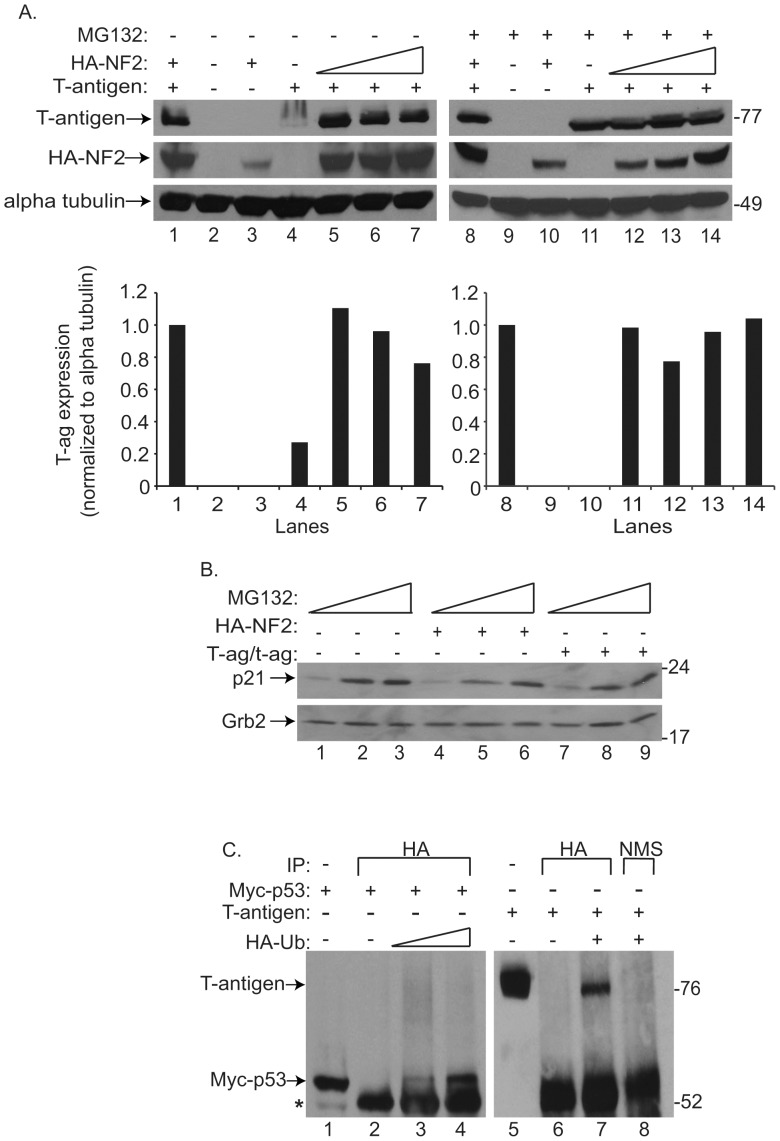
NF2 requires its FERM domain to bind and suppress T-antigen expression. To elucidate the domain of NF2 responsible for the downregulation of T-antigen protein expression, U-87 MG cells were cotransfected with T-antigen and increasing amounts of either HA-FERM or HA-ΔFERM NF2 truncation mutants (A). Immunoblotting revealed a dose-dependent decline in T-antigen expression in the presence of HA-FERM (A, lanes 1–3), but not HA-ΔFERM (A, lanes 4–6), indicating that the FERM domain is necessary for this mechanism. Band intensity representing T-antigen expression was quantified, normalized to its loading control (B). Lysates from U-87 MG cells co-transfected with T-antigen, HA-FERM, and HA-ΔFERM were utilized for Co-IP with T-antigen antibodies (C). In this assay, we demonstrate the ability of T-antigen to bind to the FERM (C, lane 5), but not ΔFERM (C, lane 10) region of NF2, further implicating FERM to be the necessary region facilitating the interaction among NF2 and T-antigen.

NF2 interacts with a wide array of cellular proteins, including the ubiquitin ligases Mdm2 and DCAF1 [22], [42] to promote the degradation of targeted proteins via the proteasome. To determine whether NF2 functions through the proteasome to regulate T-antigen expression, we exposed U-87 MG cells, transfected with T-antigen and HA-NF2 constructs, to the proteasomal inhibitor, MG132. Increasing amounts of NF2 led to a decline in T-antigen protein in samples treated with vehicle alone (Figure 4A, left), while treatment with MG132 ameliorated these affects, such that T-antigen expression was unaltered in the presence of NF2 (Figure 4A, right). Increasing amounts of MG132 led to an accumulation of p21, another protein known to be regulated by this pathway (Figure 4B), indicating the stabilization of proteins subjected to proteasomal degradation. To date, this is the first evidence of proteasomal involvement in the stability of any polyomavirus tumor antigen. A key post-translational modification involved in the targeting of a protein to the proteasome is ubiquitination. To evaluate the ability of JCV T-antigen to bind ubiquitin we conducted a Co-IP assay with T-antigen and an HA-tagged ubiquitin construct (HA-Ub). Using p53 as a protein known to bind ubiquitin, we were able to demonstrate the binding of T-antigen to ubiquitin (Figure 4C, lane 7). Together this data implicates that T-antigen may be subjected to destruction via the ubiquitin-proteasome and that NF2 can enhance this process.

### NF2, T-antigen, and p53 Interact in Human Glioblastomas

**Figure 5:**
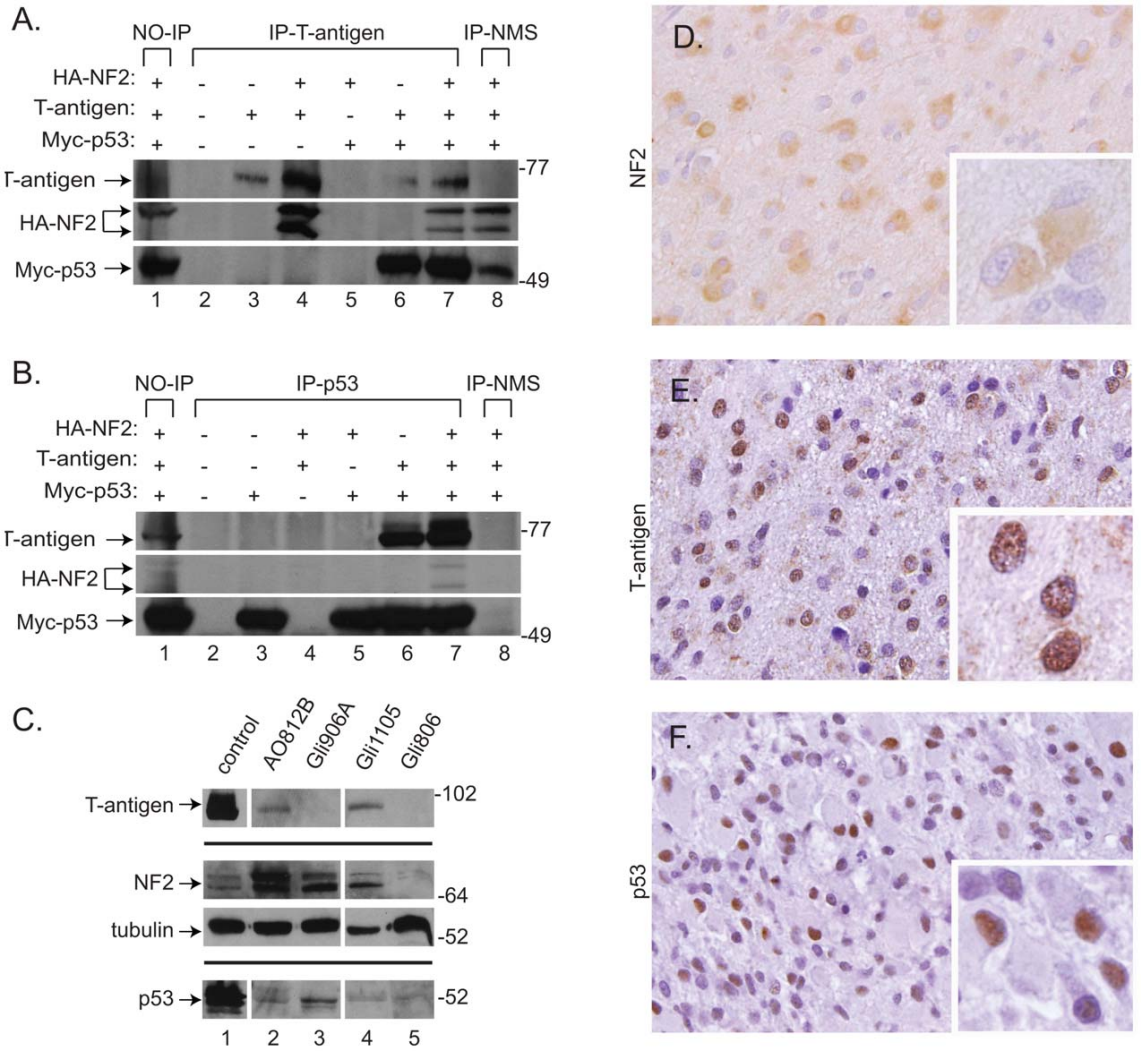
NF2, T-antigen, and p53 complex in human glioblastoma cells and are detected in high grade glioma samples. Immunoprecipitation of HA-NF2, T-antigen, and Myc-p53 proteins was performed using extracts from transiently transfected U-87 MG cells with antibodies against T-antigen (A), p53 antibody (B), or normal mouse serum as negative controls (A and B, lane 8), followed by immunoblotting to detect the presence of T-antigen, HA-NF2, or Myc-p53. As previously described, T-antigen binds NF2 (A, lane 4) and p53 (A and B, lane 6), individually. These proteins form a ternary complex (A and B, lane 7), however NF2 and p53 do not bind in the absence of T-antigen (B, lane 5). Protein lysates were prepared from fresh tissue specimens of surgically resected high grade gliomas utilized for immunoblotting revealed the presence of NF2 and p53 in an anaplastic oligodendroglioma (A, lane 2), as well as three glioblastomas (C, lanes 3–5). T- antigen was also detected in two of these tumor samples (C, lanes 2 and 4). Further, tumor tissue obtained from surgically resected high grade gliomas tumor, was paraffin embedded and used for immunohistochemistry. In the glioblastoma sample shown in Panel C, lane 4 (Gli1105), we detected cytoplasmic NF2 expression (Figure 5D). In comparable sections of the same glioblastoma sample T-antigen (E) and p53 (F) were localized to the nucleus. Panels D-F, original magnification x400; insets, original magnification, x1000; hematoxylin counterstain.

NF2, T-antigen, and p53 were previously shown to form a ternary complex in T-antigen positive tumors derived from JCV T-antigen transgenic mice [23]. Yet, it has not been determined if these proteins interact in human tumors or if NF2 and p53 can bind together in the absence of T-antigen. To this end, Co-IP assays were conducted using antibodies against both T-antigen and Myc-tagged p53 (Myc-p53) in extracts from transfected U-87 MG glioblastoma cells. T-antigen was observed to bind to both NF2 and p53, individually (Figure 5A, lanes 4 and 6 respectively) and in complex (Figures 5A and 5B, lane 7). Interestingly, binding between NF2 and p53 independent of T-antigen was not observed (Figure 5B, compare lane 5 to 7), although there is mounting evidence of a tumor suppressive synergy that exists between them [4], [43], [44].

We then assayed human high grade gliomas resected from patients for the presence of these proteins. Using immunoblotting of fresh tumor tissue, we observed the presence of significant levels of NF2 in three of four high grade glioma tumors analyzed while p53 levels were more variable (Figure 5C). In addition, we detected T-antigen protein in one anaplastic oligodendroglioma (AO812B) and one grade IV glioblastoma (Gli1105). Interestingly, high levels of NF2 and p53 were detected in another glioblastoma sample (Gli906A) in the absence of T-antigen (Figure 5C). Immunohistochemical analysis of parallel sections from one of the T-antigen positive samples (Gli1105) revealed a predominantly cytoplasmic pattern for NF2, while T-antigen and p53 were detected in the nuclei of a subset of tumor cells (Figure 5D–F). NF2 is typically examined in tumor tissues at the gene level to screen for gene deletion, i.e. LOH or mutational inactivation. However, here we examine the role of the NF2 protein as a tumor suppressor, rather than its loss of function. We were able to detect NF2 by immunohistochemistry and Western blotting in Gli1105, and as shown in Figure 5C, varying levels of NF2 are seen in the tumor tissues including some with a more prominent upper band likely representing the phosphorylated form of the protein, suggesting that total levels, as well as phosphorylation status may affect its function in human tumors.

## Discussion

NF2 is a putative tumor suppressor for glioblastoma growth, in that it is lost in many patient samples and cell lines. Reintroduction of NF2 in these cells can significantly inhibit cell proliferation, cellular transformation, and tumorigenesis [8]–[10]. While NF2 is not solely localized in the cytoplasm, the potential functions of nuclear NF2 remain unclear. Previous studies from our group indicate that the JCV regulatory protein, T-antigen, binds to NF2, but the functional significance of this interaction had not been elucidated [23]. Here, we provide evidence for a novel role of NF2 as an inhibitor of oncogenic JCV T-antigen expression in glioblastoma cells.

NF2 joins a list of only two other proteins, including p53 and SF2, that downregulate the expression of JCV T-antigen. P53 does so by binding to the JCV bidirectional promoter and preventing the transcription of T-antigen [34]. Since we could not detect NF2 interaction with the JCV promoter by ChIP assay (data not shown) nor observe an impact on promoter activity in the absence of T-antigen (Figures 1D and 1E), regulation of T-antigen by NF2 at the level of the JCV promoter appears unlikely. SF2, on the other hand, inhibits the processing of T-antigen pre-mRNA into its mature form needed for protein translation [36]. The ability for NF2 to serve in a similar capacity is also unlikely as we were unable to observe an accumulation of T-antigen pre-mRNA upon overexpression of NF2 (Figure 2C). Instead, our data suggests that NF2, specifically its tumor suppressive FERM domain, facilitates the proteasomal degradation of ubiquitin bound T-antigen. However, the accumulation of T-antigen mRNA that we observed in the presence of NF2 (Figure 2D), suggests the possibility that NF2 may also inhibit T-antigen at the level of translation. NF2 has been previously implicated to act as an inhibitor of translation [41], [45], [46]. Conversely, expression of SV40 T-antigen can activate cap-dependent protein translation [47]. Both large and small T-antigens appear to be downregulated by NF2 (Beltrami et al, unpublished observations) suggesting a common mechanism for their inhibition and, indeed, they do share a portion of their 5′ mRNA sequences and amino termini (Beltrami, unpublished observation). Further defining NF2′s potential to regulate proteasome cargo or perhaps mRNA levels, as well as identifying its cellular target proteins, are the logical next steps.

Though NF2 is traditionally viewed as a cytoplasmic scaffold, it also has tumor suppressive roles in the nucleus. NF2 has not been shown to directly regulate the cell cycle; however its presence promotes cell cycle arrest in schwannoma, primary endothelial, mesothelioma, and patient-derived meningioma cells [14], [22], [48], [49]. An intriguing protein of interest, which may link NF2 to the cell cycle, is the cyclin dependent kinase inhibitor, p21. Through its interactions with Mdm2, NF2 enhances the transcription of p21, a well established inhibitor of cell cycle progression [42], [50]. Furthermore, NF2 inhibits PAK2 from acting as a positive transcriptional regulator of the cell cycle enhancer, cyclin D1 [51]. Interestingly, T-antigen promotes the stabilization of Rac1, another cyclin D1 enhancer [52], [53]. These studies have led some to speculate that NF2 may have a novel role as an upstream cell cycle regulator [22].

While NF2 is typically a cytoplasmic protein, we have previously shown its nuclear co-localization with T-antigen in tumors derived from JCV T-antigen transgenic mice. The pattern of NF2 localization in the human high grade gliomas we examined was predominantly cytoplasmic, though examination of a more extensive set of clinical samples would be informative. In any case, many prominent nuclear proteins are regulated within the cytoplasmic compartment, p53 regulation by MDM2 is one relevant example, and a portion of T-antigen can be detected within the cytoplasm as well as in the membrane fraction of cells. A direct interaction between NF2 and p53 was not observed in the absence of T-antigen, but previous studies have suggested significant overlap between these two pathways. Interestingly, NF2 and p53 were detected in one of our glioblastoma tissue samples (Gli906A) by immunoblotting, even in the absence of T-antigen (Figure 5C). The NF2 and p53 genes are linked in the mouse genome, and loss of both genes leads to the development of multiple highly metastatic tumors [4], [44], [54] and in meningiomas, the loss of functional NF2 and p53 correlate with enhanced tumor grade [43]. At the molecular level, NF2 was shown to upregulate transcription of the p53 gene, stabilize p53 protein, and sensitize cells to p53 induced apoptosis [42]. Since NF2 and p53 do not appear to directly bind together, other cellular mediators must facilitate their interactions. One such protein may include Mdm2, the p53 ubiquitin ligase, which has been shown to bind both p53 and NF2 in isolation [42].

In our limited analysis of patient derived gliomas, we have detected both nuclear and cytoplasmic interactions among NF2 and T-antigen. There are several possibilities as to the outcome of these interactions. Nuclear NF2, in its binding to T-antigen, may prevent it from sequestering p53 and Rb and overriding their control of the cell cycle. To explore this possibility, we will investigate if NF2 can aide T-antigen expressing gliomas in regaining control of the cell cycle. In light of our recent data on the proteasomal mediated degradation of T-antigen, perhaps cytoplasmic NF2 acts as a chaperone protein to guide ubiquitin-tagged T-antigen to the proteasome. In which case, the ubiquitin ligases known to interact with NF2, namely Mdm2 and DCAF1, would be attractive candidates to explore for their ability to degrade T-antigen. Further, we were not able to determine if the p53 we detected by either immunoblotting or immunohistochemistry is wild type or mutant in these glioma samples. Previously, we have observed wild-type p53 in nervous system tumors from T-antigen transgenic mice and have demonstrated that T-antigen only interacts with wild type and not mutant p53 [23], [35], [55]. We therefore hypothesize that T-antigen would associate with wild-type p53. However, immunohistochemical detection in formalin-fixed tissue does not allow the distinction between wild-type and mutant forms of p53, therefore other means, i.e. gene sequencing or immunoprecipitation analysis would be necessary to determine the p53 status of the human tumors.

Alteration of p53 remains one of the key genetic changes in human glioblastoma and its mutation or loss has been reported in some percentage of all subtypes of glioblastomas thus far described [56]. While NF2 inactivation has been linked to schwannomas and other tumors such as mesotheliomas, loss of NF2 expression appears to be infrequent in human glioblastoma, though it has been reported in up to one third of cases [10]. Indeed, we have observed high levels of NF2 expression in high grade gliomas. However, it is important to establish whether the presence of a tumor suppressor protein such as p53 or NF2 indicates a functional protein, as the pathway may be blocked downstream and thus unable to halt cell proliferation. Should the tumor suppressor pathway be intact, it may be possible to alter the blockage and thus restore cell cycle control. Visualizing the interactive nature of these proteins in human samples provides us with a better understanding of the complex interplay among tumor suppressors and oncoproteins that shape glioblastoma growth.

## Materials and Methods

### Ethics Statement - clinical Samples

Human brain tumor tissue was obtained from surgical resections or brain biopsy under the approval of the Temple University Institutional Review Board (IRB). Written informed consent for all samples was obtained and any clinical samples presented in this study have been coded and deidentified.

### Plasmids and Cell Lines

The JCV_E_ and JCV_L_ promoter regions, from the Mad-4 strain of JC virus were cloned into separate pGL3 Basic Vectors, upstream of the *luc2P* reporter gene [57]. The expression constructs for JCV T-antigen and human p53, were created by PCR amplification and cloning into the Kpn1 and EcoR1 sites, and the EcoR1 and Not1 sites of pcDNA6/myc-His vector (Invitrogen), respectively. The HA-tagged NF2 expression construct was created by cloning into the pcDNA3 vector as described previously [23]. The T7 tagged SF2 (T7-SF2) expression construct was a gift from Ilker Sariyer [36], and the HA-tagged ubiquitin plasmid (HA-Ub) was purchased (Addgene). The U-87 MG glioblastoma cell line was obtained from the ATCC and cultured in 1x DMEM-low glucose media (GIBCO), with 10% fetal bovine serum, and 0.5 µg/µl gentamicin. The JCV T-antigen positive cell line used in this study, BSB8, was derived from JCV T-antigen transgenic mice [35], and cultured in the same media.

### Antibodies

Antibodies used for immunoblotting in this study included: HA-11 mouse monoclonal (clone 12CA5, Boehringer Mannheim), SV40 T-antigen Ab2 mouse monoclonal, which cross-reacts with JCV T-antigen, (clone pAb416, Oncogene Research Products), Myc mouse monoclonal (clone 9B11, Cell Signaling), p21 rabbit polyclonal (sc-756, Santa Cruz Biotechnologies) and alpha tubulin mouse monoclonal (clone B-5-1-2, Sigma Aldrich). For immunoprecipitation the following antibodies were utilized: SV40 T-antigen Ab2 mouse monoclonal, HA-11 mouse monoclonal, and p53 Ab-1 mouse monoclonal (clone pAb421, Wilex, Cambridge, MA). Immunohistochemistry was performed using NF2 rabbit polyclonal (C-18, Santa Cruz Biotechnologies), p53 mouse monoclonal (D07, Dako) and T-antigen mouse monoclonal (SV40 T-antigen Ab2). Patient samples were immunoblotted using NF2 rabbit polyclonal (C-18), T-antigen mouse monoclonal (SV40 T-antigen Ab2), and p53 sheep polyclonal (Ab-7, Millipore, Billerica, MA).

### Luciferase Assay

U-87 MG cells were transfected in quadruplicate with increasing concentrations of expression plasmids for HA-NF2, T-antigen, and JCV_E_ or JCV_L_ reporter plasmids using Fugene 6 transfection reagent (Roche) in Optimem media (GIBCO) and total amounts of DNA were normalized with pcDNA3.1 (empty vector). Cells were harvested 48 hours post transfection in 1× passive lysis buffer (Promega) and luciferase activity was conducted with 10 µg of protein lysates using the Luciferase Assay System kit (Promega), measured with a Femtomaster FB 12 luminometer (Zylux Co.), and quantified to give a RLU/sec/10 µg of protein.

### Immunoblotting

Whole cell extracts were prepared in 1x Promega passive lysis buffer or 0.5% NP-40, TNN buffer containing mammalian protease inhibitor cocktail (Sigma Aldrich). Proteins were separated by 9 or 12% SDS-PAGE, and transferred to nitrocellulose or PVDF membranes. Blots were blocked in 10% milk in 1× PBST, then incubated in primary antibodies for 2 hours at room temperature or overnight at 4°C. This was followed by washing, then incubation with secondary antibodies conjugated to horseradish peroxidase for detection with ECL chemiluminescence (GE Healthcare) or secondary antibodies conjugated to alkaline phosphatase for detection with CDP-Star (Perkin Elmer). Bands were quantified, using ImageJ software, and normalized to loading control.

### Co-immunoprecipitation

U-87 MG cells were transfected with expression plasmids as described in their respective figures. Forty-eight hours after transfection, whole cell extracts were prepared in 0.5% NP-40, TNN buffer containing mammalian protease inhibitor cocktail (Sigma Aldrich) and 0.1 µg/µl DNase1 (Roche). 150 µg of protein lysates were incubated overnight at 4°C with 1 µg of antibodies against either p53 (Ab-1), HA-11 tag, or T-antigen (SV40 T-antigen Ab2). Immunocomplexes were isolated by incubation with Pansorbin cells (Calbiochem) in TNN buffer, at 4°C for 2 hours. Complexes were then washed 3–4 times in TNN buffer, eluted in 2× SDS-PAGE sample buffer and subjected to immunoblot analysis.

### Proteasomal Inhibition Assay

U-87 MG cells were transfected with plasmids encoding T-antigen and increasing amounts of HA-NF2. Media was then supplemented with either 10 µM the proteasome inhibitor MG132 (Calbiochem) in DMSO or an equal amount of DMSO for 8 hours before harvesting. Whole cell extracts were prepared 48 h after transfection in TNN buffer and immunoblotted to detect T-antigen, HA-NF2, and alpha tubulin expression. In parallel, U-87 MG, untrasfected or transfected with either HA-NF2 or T-antigen plasmids, were exposed to DMSO or MG132 in DMSO (1 or 10 µM) for 8 hours. Prepared extracts were immunoblotted for the detection of p21 expression.

### Alternative Splicing PCR and qRT-PCR

RNA was extracted from U-87 MG cells transfected with either HA-NF2, T-antigen, or T7-SF2 constructs alone, or in combination, using the RNeasy kit (Qiagen), following manufacturer’s instructions. Samples were then treated with DNAse 1 (Roche) and RNAse inhibitor (Roche). Samples were subjected to PCR to verify absence of DNA contamination with primers specific for human GAPDH DNA: forward primer 5′ GAAGATGGTGATGGGATTTC 3′, reverse primer 5′ GAAGGTGAAGGTCGGAGTC 3′ at 50°C for 2 min (1×), 95°C for 10 min (1×), 95°C for 30 s/53°C for 30 s (45×), and 4°C for 1 min. When DNA was undetectable in these RNA samples, the reverse transcriptase enzyme from the Moloney Murine Leukemia Virus (Mo-MuLV RT), dNTPs, and random hexamers (Invitrogen) were used to synthesize cDNA. A portion of this cDNA was used for PCR reactions with primers to detect alternatively spliced forms of JCV T-antigen as described previously [36]: forward (4801–4780) 5′-CCTGATTTTGGTACATGGAA-3′, and reverse (4291–4313) 5′-GTGGGGTAGAGTGTTGGGATCCT-3′. The remaining cDNA was used for Q-PCR analysis of T-antigen mRNA copy number. The following primers (20 µM) and a FAM fluorophore labeled mRNA probe (10 µM) specific to JCV T-antigen were used in the PCR reaction: forward primer (4255–4274) 5′ AGTCTTTAGGGTCTTCTACC 3′, reverse primer (4408–4427) 5′ GTGCCAACCTATGGAACAG 3′, probe (4303–4327) 5′ FAM-AGTGTTGGGATCCTGTGTTTTCATCATC-BHQ1 3′. The PCR conditions for T-antigen amplification were 50°C for 2×min (1×), 95°C for 10×min (1×), 95°C for 15 s (45×), and 60°C for 1 min (1×). The standard mRNA copy number for each reaction was normalized to the average GAPDH copy number for those reaction conditions. The primers and probes used for the GAPDH PCR reactions 5′-HEX-CAAGCTTCCCGTTCTCAGCC-BHQ1–3′. All q-PCR reactions were performed in Universal Taq Man PCR Master Mix (Applied Biosystems) and amplified using the iCycler thermocycler (BioRad Laboratories).

### Immunohistochemistry

Patient tumor resection samples were fixed in formalin for 24 hour, embedded in paraffin, and sectioned into 4µm slices using a Microm HM315 (Fisher Scientific). Paraffin was melted and slides were cleared in xylenes, and rehydrated in descending concentrations of ethanol. Antigen retrieval was conducted by warming slides in citrate buffer at 95°C for 30 min. Endogenous peroxidases were quenched in a solution of methanol and 30% peroxide for 20×min. Slides were blocked in normal goat or normal horse serum, for rabbit and mouse antibodies, respectively. Primary antibodies to NF2 (C-18), p53 (D-07) and T-antigen (SV40 T-antigen Ab2) were incubated on the slides overnight in a humidified chamber. For immunohistochemical analysis, slides were incubated with the appropriate biotinylated secondary antibody, followed avidin-biotin complexes for 1hr (Vector Laboratories). Slides were developed using DAB (Sigma Aldrich), dehydrated, counter stained with hematoxylin, mounted with Permount, and visualized by bright field microscopy.
