# Correction: Neurofibromatosis Type 2 Tumor Suppressor Protein, NF2, Induces Proteasome-Mediated Degradation of JC Virus T-Antigen in Human Glioblastoma

**DOI:** 10.1371/annotation/698e3a34-84ab-4c69-8d4d-19e7c9da855b

**Published:** 2013-09-09

**Authors:** Sarah Beltrami, Emanuela Branchetti, Ilker K. Sariyer, Jessica Otte, Michael Weaver, Jennifer Gordon

The order of the titles and legends for Figures 3 and 4 is incorrect. The title and legend currently appearing with Figure 3 belong with Figure 4, and the title and legend currently appearing with Figure 4 belong with Figure 3. The figures themselves are in correct order. 

